# Dynamic calcium signals mediate the feeding response of the carnivorous sundew plant

**DOI:** 10.1073/pnas.2206433119

**Published:** 2022-07-12

**Authors:** Carl Procko, Ivan Radin, Charlotte Hou, Ryan A. Richardson, Elizabeth S. Haswell, Joanne Chory

**Affiliations:** ^a^Plant Biology Laboratory, Salk Institute for Biological Studies, La Jolla, CA 92037;; ^b^Department of Biology, Washington University in St. Louis, St. Louis, MO 63130;; ^c^NSF Center for Engineering MechanoBiology, St. Louis, MO 63105;; ^d^HHMI, Salk Institute for Biological Studies, La Jolla, CA 92037

**Keywords:** carnivorous plants, calcium signaling, mechanosensation, sundew

## Abstract

Some of the most spectacular examples of botanical carnivory—in which predator plants catch and digest animals presumably to supplement the nutrient-poor soils in which they grow—occur within the Droseraceae family. For example, sundews of the genus *Drosera* have evolved leaf movements and enzyme secretion to facilitate prey digestion. The molecular underpinnings of this behavior remain largely unknown; however, evidence suggests that prey-induced electrical impulses are correlated with movement and production of the defense hormone jasmonic acid (JA), which may alter gene expression. In noncarnivorous plants, JA is linked to electrical activity via changes in cytoplasmic Ca^2+^. Here, we find that dynamic Ca^2+^ changes also occur in sundew (*Drosera spatulata*) leaves responding to prey-associated mechanical and chemical stimuli. Furthermore, inhibition of these Ca^2+^ changes reduced expression of JA target genes and leaf movements following chemical feeding. Our results are consistent with the presence of a conserved Ca^2+^-dependent JA signaling pathway in the sundew feeding response and provide further credence to the defensive origin of plant carnivory.

Plant carnivory has long fascinated the scientific community. Indeed, during Charles Darwin’s seminal work on the subject (1), his wife Emma described him as “treating *Drosera* just like a living creature, and I suppose he hopes to end in proving it to be an animal” (2). Despite this interest, our molecular understanding of the animal-like ability to sense and capture prey remains largely incomplete, in part due to the genetic intractability of working with these nonmodel plants.

Sundew species typically grow as small rosettes. Their most striking feature is the presence of hairs—or so-called tentacles—on the adaxial leaf surface. Most tentacles secrete a drop of sticky mucilage from a glandular tip (the tentacle head), to which animal prey adheres. Stimuli from the captured animal generate action potentials along the tentacle and cause oscillations in membrane potential of the local leaf blade (3, 4). These electrical signals are followed by rapid movement of the stimulated tentacles and others nearby toward the leaf center, and later by leaf blade inflection over the animal, creating an “outer stomach” (Movie S1) (1, 4). Concomitant local increases in jasmonic acid (JA) may alter gene expression necessary for digestion and/or contribute to inflection (5).

Electrical signaling is not unique to carnivorous plants. In *Arabidopsis thaliana* (*Arabidopsis*), mechanical wounding by herbivorous insects generates systemic electrical signals that coincide with waves of increasing cytoplasmic Ca^2+^ concentration ([Ca^2+^]_cyt_) that drive JA production (6, 7). JA involvement in both plant wounding/defense and carnivory has led to the hypothesis that carnivory evolved from the JA insect defense pathway, at least in the Droseraceae (8). As such, Ca^2+^ changes might be important for prey recognition in sundews. Indeed, in the related Venus flytrap, *Dionaea muscipula*, Ca^2+^ changes in the bilobed leaf blade correlate with leaf movement for prey capture (9). The snapping movement of the Venus flytrap leaf directly follows touch stimulation and occurs within 100 ms (10). By comparison, sundew leaf blade inflection occurs over several hours, suggesting a greater temporal separation between movement and cellular signaling events in this species. Furthermore, sundew leaf inflection—like the slow, hermetical sealing of the Venus flytrap leaf following its initial rapid closure—is affected by both mechanical and chemical cues (1, 11). To date, a direct test of the involvement of Ca^2+^ signaling in sundew carnivory and its link to JA has been lacking. Likewise, whether and how [Ca^2+^]_cyt_ dynamics in carnivorous plants are altered by chemical as well as mechanical cues remains to be explored, despite both being important for carnivorous behavior. Here, we address this knowledge gap.

## Results

To image [Ca^2+^]_cyt_, we generated transgenic *Drosera spatulata* plants expressing the Ca^2+^ reporter GCaMP3 (12) using biolistics, the first reported transformation for this species that we know of. These plants displayed pleiotropic defects (SI Appendix). Nevertheless, many leaves responded to feeding, allowing us to explore [Ca^2+^]_cyt_ changes in these plants.

**Fig. 1.**
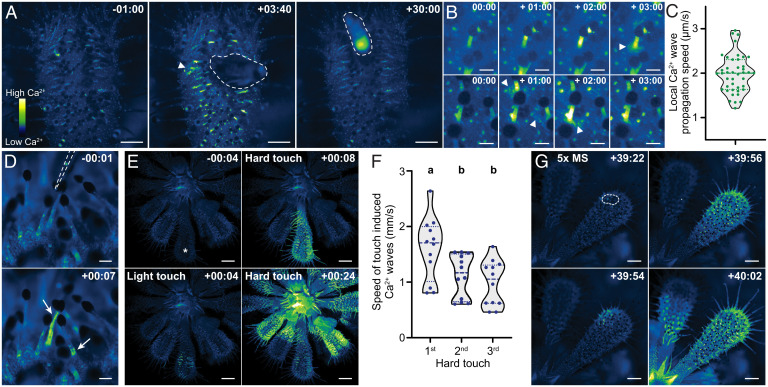
Prey-associated stimuli induce dynamic Ca^2+^ responses in carnivorous sundew plants. (*A*) Ca^2+^ response of a transgenic *D. spatulata* leaf expressing a GCaMP3 reporter to a live fly (dashed line) added at time point 00:00. (*B*) Ca^2+^ response of tentacles that either were (*Top*) or were not (*Bottom*) in direct contact with the fly. (*C*) Speed of Ca^2+^ waves spreading from the tentacle base in response to fly presence (arrowheads in *A* and *B*). Dashed line, median; dotted lines, quartiles. (*D*) Ca^2+^ response of two tentacles (arrows) touched and bent at time point 00:00 with a glass probe (dashed line). (*E*) Ca^2+^ response of a sundew plant manually touched with a glass Pasteur pipette. The indicated leaf (asterisk) was either touched (time point 00:00) lightly with a single downward movement or hard with lateral movement. (*F*) Speed of systemic Ca^2+^ waves induced by three sequential (4-min intervals) hard touches. Dashed line, median; dotted lines, quartiles. Statistics, one-way ANOVA with post hoc Tukey test (*P* < 0.05). (*G*) Ca^2+^ response to 2-µL 5× MS salt solution added to a small area of the leaf (dashed line). All timestamps are min:s. (Scale bars: 1 mm [*A* and *G*], 0.2 mm [*B*], 0.1 mm [*D*], 2 mm [*E*].).

Feeding of live *Drosophila melanogaster* flies to leaves caused dynamic [Ca^2+^]_cyt_ changes. Most prominent was increased GCaMP3 fluorescence and flickering in tentacles, observed in 19/20 experiments (Fig. 1*A* and Movie S2). The increase in GCaMP3 intensity decreased over time and was gone within 30 min, even if the insect remained trapped and continued struggling (*n* = 8). Tentacles under or in close contact to the fly sent a Ca^2+^ wave from the tentacle head down to the base, followed by relatively low-intensity, slow-moving waves that propagated outward from the base (Fig. 1*B*, *Top* and *C* and Movie S3; *n* = 14/20). These changes correlate with the top-down propagation of action potentials in the tentacle and oscillations in leaf blade membrane potential following head stimulation recorded by others (4, 13). In addition, in nine of our experiments, tentacles close to the fly but seemingly not in direct contact also exhibited increased GCaMP3 intensity, followed by the spread of slow-moving local Ca^2+^ waves (Fig. 1*B*, *Bottom* and Movie S4).

Insect prey can elicit mechanical and chemical signals, and both can alter electrical activity of tentacles (3). As observed by Darwin (1), dead prey induced less leaf inflection. In our hands, 83% of live fly-fed wild-type leaves inflected beyond a 90° angle after 6 h, whereas only 33% did with a dead fly (*n* = 30, both treatments; *P* < 0.005, Fisher’s exact test). We therefore assessed the effect of mechanical touch on [Ca^2+^]_cyt_. When bent, 84% (52/62) of tentacles showed changes in GCaMP3 intensity, with Ca^2+^ signal increasing throughout the stalk or near the base (Fig. 1*D* and Movie S5). Similarly, a gentle manual touch of a glass probe onto the leaf caused transient GCaMP3 increases similar to those caused by live prey (Fig. 1*E* and Movie S6; *n* = 8). However, hard touch with some lateral probe movement initiated one or more intense Ca^2+^ waves that propagated rapidly to distal leaves (Fig. 1*E* and Movie S7). The average speed of the first systemic wave was 1.6 mm/s, similar to those in *Arabidopsis* following wounding (7), but not as fast as those in Venus flytrap responding to touch (9). Subsequent waves induced by further touch were slower (Fig. 1*F*). These Ca^2+^ waves might be associated with mechanical wounding, which elicits systemic electrical impulses in sundew that do not cause local leaf inflection (4).

To assess the effect of chemical stimuli, we fed salts to the leaves. A 5-μL drop of 5× Murashige and Skoog basal salts with vitamins (MS) solution placed on the leaf blade caused leaf inflection of 90° or more in 15 of 30 leaves in a 12-h period. By contrast, 0/30 water-treated controls inflected (*P* < 0.005, Fisher’s exact test), suggesting that inflection is due to salt treatment and not mechanical placement of the liquid droplet onto the leaf. Over 3 h, salt treatment (2 μL 5× MS salt solution) also induced one or more systemic Ca^2+^ waves in four of five treated leaves (Fig. 1*G* and Movie S8). These occurred 2 min to 70 min following treatment, with a speed similar to those induced by hard touch (first wave mean, 1.7 mm/s), and were not observed in water-treated controls (*n* = 5).

**Fig. 2.**
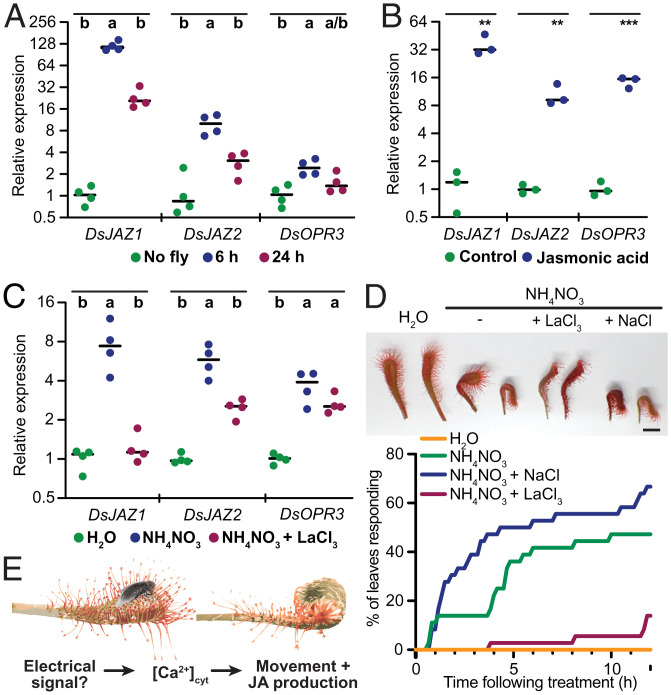
Changes in [Ca^2+^]_cyt_ are required for chemical feeding responses. (*A–C*) Relative expression (ΔΔC_t_) of *D. spatulata* gene transcripts *DsJAZ1*, *DsJAZ2*, and *DsOPR3* measured by qRT-PCR in (*A*) attached leaves fed with a live fly; (*B*) detached leaves treated for 12 h with 500 µM JA; or (*C*) detached leaves treated for 6 h with water (mock), 100 mM NH_4_NO_3_, or 100 mM NH_4_NO_3_ + 20 mM LaCl_3_. Dots, biological replicates; lines, averages. Statistics, (*A* and *C*) one-way ANOVA with post hoc Tukey test (*P* < 0.05) within indicated groups; (*B*) unpaired *t* test within indicated groups (**P < 0.005 and ***P < 0.0005). (*D*) Effect of LaCl_3_ or NaCl (20 mM) on the NH_4_NO_3_ (100 mM) induced detached leaf blade inflection. (*Top*) Example leaf response after 12 h. (Scale bar, 5 mm.) (*Bottom*) Percentage of leaves that inflected 90° or greater over time (*n* = 36 each treatment, scored at 10-min intervals). The experiments in *C* and *D* included pretreatments; see SI Appendix for details. (*E*) Model of the carnivorous response in sundew.

Finally, to determine whether [Ca^2+^]_cyt_ changes in response to feeding might be associated with JA, we tested the effect of the Ca^2+^ channel blocker La^3+^ on JA-dependent gene expression. Transcripts showing homology to *Arabidopsis* JA target genes *JASMONATE-ZIM-DOMAIN PROTEIN 1* and *2* (*JAZ1* and *JAZ2*) and *OXOPHYTODIENOATE-REDUCTASE 3* (*OPR3*) (14) were induced in sundew leaves by fly feeding and exogenous JA application (Fig. 2*A* and *B*). To test the effect of La^3+^, we performed chemical bath treatments of detached leaves, similar to methods used by Darwin (1). Because LaCl_3_ caused some precipitation in MS salts, we instead treated leaves with NH_4_NO_3_, a component of MS salts that, alone, elicits feeding behavior (1). Similar to fly feeding, we found that NH_4_NO_3_ induced all three JA target genes (Fig. 2*C*). Strikingly, the addition of LaCl_3_ reduced induction, with statistical significance for two of the three genes tested (Fig. 2*C*). Lastly, LaCl_3_ also slowed leaf blade inflection to NH_4_NO_3_, whereas NaCl at the same concentration had the opposite effect (Fig. 2*D*). Together, these results suggest that Ca^2+^ changes are necessary for full behavioral and transcriptional responses of the leaf to feeding.

## Discussion

Here, we report prey capture–associated dynamic cytoplasmic Ca^2+^ changes in carnivorous sundew plants. These changes are both local and systemic in nature and precede leaf movements. How the plant interprets and integrates these and other Ca^2+^ signals to generate an appropriate response remains an outstanding question.

Interestingly, leaf trichomes of *Arabidopsis* also elicit touch-induced Ca^2+^ signals. For example, trichomes may sense mechanical stimulation from falling rain droplets to defensively prepare against rain-associated pathogen invasion (15), or detect touch from neighboring plants, causing leaf hyponasty (16). It is tempting to draw evolutionary parallels between these trichomes and sundew tentacles, both of which can affect leaf movements in response to mechanical cues.

Finally, our results further advance the notion that carnivory in the Droseraceae evolved from the JA insect defense pathway (8). Our finding that JA-responsive gene induction is at least partly dependent on Ca^2+^ signals is consistent with a model whereby Ca^2+^ oscillations alter JA production (Fig. 2*E*). With transformation methods of carnivorous plants improving, the field is now ripe for further molecular investigations into the charismatic predatory behavior of these plants.

## Materials and Methods

Callus generated from sundew leaf explants was bombarded with gold particles carrying a 2×*35S* promoter:*GCaMP3* plasmid. From this, a transgenic reporter line was regenerated and clonally propagated in tissue culture before moving to soil. Clonal Ca^2+^ reporter plants were imaged with fluorescent dissecting microscopes. All other experiments used wild-type plants. For qRT-PCR, SYBR Green-based methods were used. See SI Appendix for details.

## Supplementary Material

Supplementary File

Supplementary File

Supplementary File

Supplementary File

Supplementary File

Supplementary File

Supplementary File

Supplementary File

Supplementary File

## Data Availability

All study data are included in the article and/or supporting information. Plant strains are available on request from the corresponding author.

## References

[1] C. R. Darwin, Insectivorous Plants (John Murray, London, 1875).

[2] H. E. Litchfield, Emma Darwin, Wife of Charles Darwin (Cambridge University Press, 2010).

[3] S. E. Williams, B. G. Pickard, Receptor potentials and action potentials in *Drosera* tentacles. Planta 103, 193–221 (1972). 24481555 10.1007/BF00386844

[4] M. Krausko , The role of electrical and jasmonate signalling in the recognition of captured prey in the carnivorous sundew plant *Drosera capensis*. New Phytol. 213, 1818–1835 (2017). 27933609 10.1111/nph.14352

[5] Y. Nakamura, M. Reichelt, V. E. Mayer, A. Mithöfer, Jasmonates trigger prey-induced formation of ‘outer stomach’ in carnivorous sundew plants. Proc. Roy. Soc. B 280, 20130228 (2013). 10.1098/rspb.2013.0228 PMC3619512 23516244

[6] S. A. R. Mousavi, A. Chauvin, F. Pascaud, S. Kellenberger, E. E. Farmer, GLUTAMATE RECEPTOR-LIKE genes mediate leaf-to-leaf wound signalling. Nature 500, 422–426 (2013). 23969459 10.1038/nature12478

[7] M. Toyota , Glutamate triggers long-distance, calcium-based plant defense signaling. Science 361, 1112–1115 (2018). 30213912 10.1126/science.aat7744

[8] A. Pavlovič, A. Mithöfer, Jasmonate signalling in carnivorous plants: Copycat of plant defence mechanisms. J. Exp. Bot. 70, 3379–3389 (2019). 31120525 10.1093/jxb/erz188

[9] H. Suda , Calcium dynamics during trap closure visualized in transgenic Venus flytrap. Nat. Plants 6, 1219–1224 (2020). 33020606 10.1038/s41477-020-00773-1

[10] Y. Forterre, J. M. Skotheim, J. Dumais, L. Mahadevan, How the Venus flytrap snaps. Nature 433, 421–425 (2005). 15674293 10.1038/nature03185

[11] J. Jakšová , Taste for protein: Chemical signal from prey stimulates enzyme secretion through jasmonate signalling in the carnivorous plant Venus flytrap. Plant Physiol. Biochem. 146, 90–97 (2020). 31734521 10.1016/j.plaphy.2019.11.013

[12] L. Tian , Imaging neural activity in worms, flies and mice with improved GCaMP calcium indicators. Nat. Methods 6, 875–881 (2009). 19898485 10.1038/nmeth.1398 PMC2858873

[13] S. E. Williams, B. G. Pickard, Properties of action potentials in *Drosera* tentacles. Planta 103, 222–240 (1972). 24481556 10.1007/BF00386845

[14] H. S. Chung , Regulation and function of Arabidopsis *JASMONATE ZIM*—Domain genes in response to wounding and herbivory. Plant Physiol. 146, 952–964 (2008). 18223147 10.1104/pp.107.115691 PMC2259048

[15] M. Matsumura , Mechanosensory trichome cells evoke a mechanical stimuli-induced immune response in *Arabidopsis thaliana*. Nat. Commun. 13, 1216 (2022). 35260555 10.1038/s41467-022-28813-8 PMC8904797

[16] C. K. Pantazopoulou , Mechanodetection of neighbor plants elicits adaptive leaf movements through calcium dynamics. bioRxiv [Preprint] (2022). https://www.biorxiv.org/content/10.1101/2022.01.28.478192v1. Accessed 30 March 2022. 10.1038/s41467-023-41530-0 PMC10511701 37730832

