# Supplementary material for: Dynamic calcium signals mediate the feeding response of the carnivorous sundew plant
Source: Proc Natl Acad Sci U S A. 2022 Jul 12;119(30):e2206433119. doi: 10.1073/pnas.2206433119 (PMC9335315; doi:10.1073/pnas.2206433119)
Supplement: Supplementary File [file pnas.2206433119.sapp.pdf]

PNAS

***SI Appendix* for**

Dynamic calcium signals mediate the feeding response of the carnivorous sundew plant

Carl Procko*, Ivan Radin*, Charlotte Hou, Ryan A. Richardson, Elizabeth S. Haswell, and Joanne Chory.

This file includes:

Supporting Materials and Methods
Supporting Movie legends
Supporting References

Supporting Materials and Methods

Plant propagation and transformation

Drosera spatulata var. *lovellae* seeds (A.K. Carnivores, HI) were surface-sterilized and germinated on propagation medium (1/3 MS salts and vitamins [Caisson Labs, catalog # MSP09], 3% sucrose, 1:1000 plant preservative mixture [Caisson Labs], 300 µg/mL carbenecillin, 4.3 g/L Gellex [Caisson Labs], pH 5.6). Tissue culture plates were incubated at 25°C under cycling 16 h light 8 h dark conditions, PAR 30 µmol/m^2/s. Clonally-propagated plants generated from a single seed were used in all subsequent experiments (strain name: coral). For experiments, clonal rosettes were moved to wet fine-grain sphagnum peat and grown in a greenhouse (~25°C) for >1 month prior to scoring. Wild-type plants were used for all experiments, except for Ca^{2+}-imaging experiments using the transgenic reporter line.

To generate the reporter line, cut leaves were placed on propagation medium with hormones (1 mg/L 6-benzyl-aminopurine and 0.5 mg/L kinetin [Sigma]). After 1-2 months, callus was cut from the leaf explants and bombarded with 1 µm gold particles coated with plasmid pCP18.40H (see below) at 1350 psi using a Bio-Rad PDS-1000/He Biolistic Particle Delivery System. Callus was returned to hormone-supplemented medium and incubated in the dark for 7 d before transferring back to cycling light conditions and hormone-supplemented medium with 10 µg/mL hygromycin. During this time, a single GCaMP3 positive sector of callus was observed by fluorescence and propagated. After ~4 months, hormones were removed to encourage rosette growth. Daughter rosettes were then continually split and propagated over 3-4 months until non-mosaic plants were obtained, as indicated by the presence of GCaMP3 fluorescence throughout all tissue. These were clonally expanded on propagation medium without selection for >3 months before moving to sphagnum peat and greenhouse-cultivation for >6 months prior to imaging. Reporter plants were noticeably smaller than non-transgenic controls, had reduced mucilage production, senesced earlier, and had reduced response to chemical feeding and JA application (specifically, after 6 h, 67% and 53% of wild-type leaves showed a leaf inflexion angle of 90° or greater when fed with a 10 µL drop of 5% milk solution [Carnation instant non-fat dry milk] or 500 µM JA solution [Sigma], respectively, compared to 53% and 13% of transgenic reporter leaves; N = 30 all treatments). Leaves appearing phenotypically healthy were chosen for imaging.

Molecular Cloning

GCaMP3 cDNA was PCR amplified using primers:
5’-GGGGACAAGTTTGTACAAAAAAGCAGGCTccATGGGTTCTCATCATCATCATCATC-3’
5’-GGGGACCACTTTGTACAAGAAAGCTGGGTcTTACTTCGCTGTCATCATTTGTACAA-3’
This was recombined with Gateway (Thermo Fisher Scientific) vector pDONR 221 to create pCP18.39. Other plasmids included pDONR P4-P1R recombined with a 2x35S promoter element, and pDONR P2R-P3 carrying a 25 bp random sequence (see (1)). These were recombined with binary vector pH7m34GW to create 2x*35S* promoter:*GCaMP3* reporter plasmid pCP18.40H.

Imaging

To minimize the influence of environmental changes on Ca^{2+} dynamics, plants used for imaging (except for Fig. 1G which were kept in a greenhouse as described above) were cultured in a growth chamber at a stable temperature of 25°C and relative humidity of 75% and under 16/8 h light/dark cycle with 100 μmol/m^2/s light intensity. Pots were kept in standing double deionized water and covered with a transparent dome to maintain high humidity. These conditions ensured the production and maintenance of mucilage on tentacles.

Plants were imaged using either a Leica THUNDER Imager Model Organism Microscope MC205FA with 1x/0.06 objective with varying zoom, ET GFP fluorescence cube (525 nm emission), and Leica-DFC9000GT-VSC11006 camera (used for Fig. 1A-B, D-E) or ZEISS Axio Zoom.V16 Microscope with Objective Plan Z 1.0x/0.25 FWD 60mm, Zeiss filter set 38 HE, and Zeiss Axiocam 705 mono camera (used for Fig. 1G). Prior to imaging, plants were adjusted to the conditions of the imaging facility (dark and slightly lower temperature) for 30-60 min. For fly (*Drosophila melanogaster*) feeding experiments in Fig. 1A-B plants were kept and imaged in standing water under a transparent dome to ensure maintenance of high humidity and mucilage on leaves. Flies were temporarily anaesthetized by cold, and a single fly gently placed on the middle of the leaf immediately prior to imaging. For the time lapse shown in Movie S1, an Olympus TG-6 camera was used. For other experiments in Fig. 1D-E, G, plants were uncovered and hence at lower humidity. Individual tentacles (Fig. 1D) were bent with a dull fine glass pipette (approximately 30-40 μm thick at the tip) attached to a micromanipulator, while the whole leaves were touched by hand with a fire-polished (sealed tip) glass Pasteur pipette. For chemical feeding experiments in Fig. 1G, 2 μL of 5x MS salts with vitamins (Caisson Labs catalog #MSP09) were added with a pipette just prior to the start of imaging. Extended high salt treatments were detrimental to plant health, and, as such, treated leaves were removed and discarded after imaging.

Time-lapse images were exported as TIFF files and processed with Fiji/ImageJ (2). "Green fire blue" lookup table was used for images in Fig. 1 and supplemental videos and display intensity ranges were adjusted to facilitate clarity. The speed of both local (Fig. 1C; 5 independent experiments) and systemic (Fig. 1F; 4 independent experiments) Ca^{2+} waves were determined by manually measuring the distance the wavefront traversed over a specific time period. To ensure consistency, the propagation speed of the systemic waves was measured in the petiole and the base of the touched leaf.

Bath treatments and quantitative reverse transcriptase- (qRT-)PCR

Bath treatments for qRT-PCR and leaf inflexion assays were used to facilitate chemical application to the entire leaf surface. These were performed using 12 well plates (Falcon catalog # 353043) with 4 mL of the indicated solution per well. Leaves were cut from wild-type plants at the base of the petiole and placed adaxial-side down into the well. Three leaves were added to each well, and the assay performed at ambient

temperature. For the $LaCl_3$ experiments shown in Fig. 2*C* and *D*, detached leaves were also pre-treated for 1 h with H_2O (H_2O and NH_4NO_3 treatments), 20 mM NaCl (NH_4NO_3 + NaCl) or 20 mM $LaCl_3$ (NH_4NO_3 + $LaCl_3$) immediately prior to moving the detached leaves to the final assay solution (time 0). Results are shown for leaves of plants growing in late summer/autumn months, which had a stronger inflexion behavior than those growing in late winter/early spring. For qRT-PCR, 4 leaves were pooled for each sample, and collected in triplicate or quadruplicate. Amplification was performed on a Bio-Rad CFX384 Real-Time System. Reaction concentrations were: 0.2 µM each primer, 1 mM dNTPs, 10 U/mL Phusion High-Fidelity DNA Polymerase (New England BioLabs catalog # M0530), 1x Phusion HF buffer, and 1x SYBR Green (10x stock made with 20 µL 10,000x SYBR Green concentrate [Invitrogen catalog # S7563], 200 µL Tween 20, 2 mL of 10 mg/mL BSA stock, 10 mL DMSO and 7.78 mL H_2O). Cycling conditions were 95°C 10 s, 60°C 20 s and 72°C 20 s. qRT-PCR primers were designed by searching the *D. spatulata* transcriptome and genome sequences (3) using BLAST tools (NCBI) for potential transcripts or parts thereof showing homology to Arabidopsis JA-target genes *JAZ1* (*D. spatulata* transcript ID Ds_00008226-RA), *JAZ2* (a region located on *D. spatulata* genomic sequence contig tig00014108_pilon at position 621222-621730), and *OPR3* (Ds_00016024-RA) (3). These were normalized against a reference transcript with homology to *GLYCERALDEHYDE-3-PHOSPHATE DEHYDROGENASE* (*GAPDH*) *OF PLASTID 2* (GAPCP-2), a *GAPDH* isoform (Ds_00016334-RA). For the data analysis, the C_t values for technical replicates were averaged and corrected for primer efficiency using the formula C_t=C_{t_raw}*(log(E)/log(2)), with E=(E%*0.01)+1. The corrected C_t values for each sample were normalized (ΔC_t) to *GAPDH.* The relative expression ($\Delta\Delta C_t$) for each biological replicate was calculated using the formula $\Delta\Delta C_t$=2^-($\Delta C_{t\text{-Sample}}$ – Average $\Delta C_{t\text{-control}}$). Primers used: *DsGAPDH*, 5’-GGAAGCAAGACAAGGGTAGG-3’ and 5’-GTAACTTTCATCTGTTTCCCGT-3’; *DsJAZ1*, 5’-ACGATGAATTTGTTCCCACTG-3’ and 5’-GCATTTAGCCTGTCCTTTCTC-3’; *DsOPR3*, 5’-TCTCATCCTGTGTATCAGCC-3’ and 5’-AGTCTATGCCATCGTTGAACTC-3’; and *DsJAZ2*, 5’-GAACAAGTCATCGAGCTTCTC-3’ and 5’-CCTTCCTCACTATAGGCATTTCC-3’. Graphs in Fig. 2A-C are depicted with logarithmic (base 2) Y-axes.

Statistical analysis

Statistical analysis was performed in GraphPad Prism 9. D'Agostino & Pearson and Shapiro-Wilk tests were used to test data normality. The same software package was used to generate all graphs.

Supporting Movie Legends

Movie S1
Time lapse showing leaf blade inflexion response of the sundew *D. spatulata* following feeding with a *D. melanogaster* fly.

Movie S2
Example of a transgenic *D. spatulata* sundew leaf expressing the GCaMP3 reporter protein responding to a live *D. melanogaster* fly.

Movies S3
Example of Ca^{2+} response in a sundew tentacle stimulated by a moving fly. Note increase in GCaMP3 intensity in the tentacle, as well as the presence of a slow-moving wave propagating out from the tentacle base.

Movies S4
Example of Ca^{2+} response in a sundew tentacle in close proximity but not in contact with moving fly.

Movie S5
Example of Ca^{2+} signals in individual tentacles bent with a glass probe.

Movie S6
Ca^{2+} signals on a sundew leaf when gently touched with a fire-polished Pasteur pipet.

Movie S7
Example of a fast-moving, systemic Ca^{2+} wave induced by a hard touch stimulus on the sundew leaf.

Movie S8
Example of a fast-moving, systemic Ca^{2+} wave induced by a chemical stimulus on the sundew leaf (2 µL 5x MS salts).

Supporting References

1. C. Procko *et al.*, The epidermis coordinates auxin-induced stem growth in response to shade. *Genes Dev* **30**, 1529-1541 (2016).
2. J. Schindelin *et al.*, Fiji: an open-source platform for biological-image analysis. *Nature Methods* **9**, 676-682 (2012).
3. G. Palfalvi *et al.*, Genomes of the Venus Flytrap and Close Relatives Unveil the Roots of Plant Carnivory. *Current Biology* **30**, 2312-2320.e2315 (2020).
